# Hearing performance as a predictor of postural recovery in cochlear implant users^☆^

**DOI:** 10.1016/j.bjorl.2016.01.002

**Published:** 2016-03-31

**Authors:** Mario Edvin Greters, Roseli Saraiva Moreira Bittar, Signe Schuster Grasel, Jeanne Oiticica, Ricardo Ferreira Bento

**Affiliations:** a Pontifícia Universidade Católica de Campinas (PUC–Campinas), Departamento de Otorrinolaringologia, Campinas, SP, Brazil; b Universidade de São Paulo (FMUSP), Faculdade de Medicina, Departamento de Otorrinolaringologia, São Paulo, SP, Brazil

**Keywords:** Dizziness, Balance, Hearing loss, Posturography, Auditory evoked potentials, Cochlear implant, Tontura, Equilíbrio, Perda auditiva, Posturografia, Potenciais evocados auditivos, Implante coclear

## Abstract

**Objective:**

This study aimed to evaluate if hearing performance is a predictor of postural control in cochlear implant (CI) users at least six months after surgery.

**Methods:**

Cross-sectional study including (CI) recipients with post-lingual deafness and controls who were divided into the following groups: nine CI users with good hearing performance (G+), five CI users with poor hearing performance (G−), and seven controls (CG). For each patient, computerized dynamic posturography (CDP) tests, a sensory organization test (SOT), and an adaptation test (ADT) were applied as dual task performance, with first test (FT) and re-test (RT) on the same day, including a 40–60 min interval between them to evaluate the short-term learning ability on postural recovery strategies. The results of the groups were compared.

**Results:**

Comparing the dual task performance on CDP and the weighted average between all test conditions, the G+ group showed better performance on RT in SOT4, SOT5, SOT6, and CS, which was not observed for G− and CG. The G− group had significantly lower levels of short-term learning ability than the other two groups in SOT5 (*p* = 0.021), SOT6 (*p* = 0.025), and CS (*p* = 0.031).

**Conclusion:**

The CI users with good hearing performance had a higher index of postural recovery when compared to CI users with poor hearing performance.

## Resumo

**Objetivo:**

O presente estudo teve por objetivo avaliar se o desempenho auditivo é preditor de controle postural em usuários de IC pelo menos 6 meses após a cirurgia.

**Método:**

Estudo transversal consistindo em recipientes de implante coclear (IC) com surdez pós-lingual e controles, que foram divididos nos seguintes grupos: nove usuários de IC com bom desempenho auditivo (G+), cinco usuários de usuários de IC com desempenho auditivo insatisfatório (G-) e sete controles (GC). Aplicamos o teste de posturografia dinâmica computadorizada (PDC), teste de organização sensitiva (TOS) e teste de adaptação (TAd) como desempenho de dupla tarefa, primeiro teste (PT) e reteste (RT) no mesmo dia, com intervalo de 40-60 minutos entre testes, com o objetivo de avaliar a capacidade de aprendizado em curto prazo nas estratégias de recuperação postural. Comparamos os resultados dos testes.

**Resultados:**

Comparando o desempenho de dupla tarefa no teste PDC e a média ponderal entre todas as condições de teste, o grupo G+ demonstrou melhor desempenho no RT nos TOS4, TOS5, TOS6 e EC, o que não foi observado para os grupos G- e GC. O grupo G- obteve níveis significantemente mais baixos de capacidade de aprendizado em curto prazo *vs.* outros dois grupos no TOS5 (p = 0,021), TOS6 (p = 0,025) e EC (p = 0,031).

**Conclusão:**

Usuários de IC com bom desempenho auditivo tiveram índice melhor de recuperação postural, quando comparados a usuários de IC com desempenho auditivo insatisfatório.

## Introduction

Cognitive resources from the right cerebral hemisphere significantly contribute to postural control. The assessment of adults with vestibular disorders showed that implementation of mental tasks during the performance of the platform test can both increase or reduce subject oscillations and body balance control.^1^ Working memory, short-term memory, and executive function are frequently and more highly disturbed in those stricken by bacterial meningitis.^2^ In a previous study performed at the present clinic with cochlear implant (CI) users (study submitted), a significant increase was found in P3 latency in patients with post-lingual deafness due to meningitis, suggesting an impairment of cognitive function.

Sensory input coming from hearing, vision, vestibular, and proprioceptive systems is processed in the central nervous system (CNS), resulting in spatial orientation, image fixation on the retina, and balance control.^3^ Postural adjustments necessary for balance stability result from complex motor responses, learned motor tasks, and proactive and feedforward postural strategies.^4^

Balance control is the ability to maintain body movement within the base of support without falling. It is a complex control process that depends on mainly two distinct and interdependent systems: (1) the gaze stabilization system, which maintains gaze direction of the eyes and visual acuity during head and body movements, and (2) the postural stabilization system, which keeps the body in balance while standing and moving in daily life. They are distinct because they rely on inputs from different senses, motor reactions from different parts of the body, and are mediated by different brain pathways. They are also interdependent because gaze stability is not possible unless the body is also stable, and because accurate vision is a critical sensory input for postural control. Therefore, balance depends on various sensory inputs which inform the brain about body position related to the environment. It is a highly complex network that includes numerous synaptic pathways, non-synaptic pathways, and their intersections. The brain must analyze and plan the motor responses and movements necessary for postural stabilization.^5^

At this moment cognitive function is required for the accurate sensory integration to achieve a suitable body balance control. The following are part of cognition: (1) the sensory input integration of the brain map composed of movement strategies learned throughout life, (2) appropriate latencies of postural responses, and (3) the ability to plan and execute movement patterns necessary for controlling the center of the body mass. The cortex must process and integrate these functions to be aware of the risk and the task being performed. Thus, balance control is influenced by cognitive factors like attention, motivation, memory, and intent. In normal individuals, the whole system works in harmony and is processed automatically, but may require voluntary control when there is imbalance at some point of the track.

The acquisition of new motor skills is related to CNS structures responsible for memory and learning.^6^ Among structures required for that task are areas of the medial temporal lobe, particularly the hippocampus, motor cortical regions (striatum sensory motor cortex), cerebellar cortex and nucleus, parietal cortex, and frontal association areas.^4^, ^7^, ^8^ Among the various injuries that may affect the postural reactions, those affecting the hippocampus may limit vestibular compensation.^8^ Likewise, speech understanding and interpretation depend on learning and memory.

The Department of Otolaryngology at Hospital das Clínicas University of São Paulo School of Medicine (HC-FMUSP) performs dozens of CI surgeries on deaf patients every year; all of them have routine auditory evoked potential tests. Based on the authors’ clinical experience (study submitted), it was observed that subjects with poor word discrimination had a prolonged P3 latency in the cognitive auditory evoked potential test. It was shown that CI users with poor hearing performance (≤80% speech recognition in open-set sentences), particularly those deaf due to meningitis, show longer P3 latencies as compared to deaf patients with good hearing performance after CI (study submitted). Thus, the P3 test seems to be useful to detect impairment of central sensory integration after auditory stimulation. The literature demonstrates intersections between auditory, vestibular, learning, and memory pathways. There is evidence of hearing tests as predictors of falls^6^ and evidence that computerized dynamic posturography (CDP) shows secondary changes due to noise-induced hearing loss.^9^

Both auditory memory and balance depend on the integrity of CNS structures. So, it would be plausible to assume that subjects suffering from central auditory pathway impairment could also have trouble in tasks of postural recovery involving cognition. In order to evaluate the relationship between auditory and vestibular cognitive distress, the authors developed a method to assess postural recovery (short-term memory, a task that depends directly on proper functioning of the hippocampus) in subjects with and without good hearing performance after CI surgery.

## Objectives

This study aimed to evaluate whether hearing performance is a predictor of postural control in CI users at least six months after surgery.

## Methods

### Sample

Cross-sectional study conducted between 2008 and 2011, including 111 CI users with post lingual deafness from the CI Group of HC-FMUSP. The following variables were assessed: gender, age, cause of deafness, and CI electrode type. The study was carried out according to the guidelines of the Ethics Committee of the University of São Paulo School of Medicine and was approved in 2007 under protocol No. 1059/07.

### Selection criteria

Patients were considered eligible according to the following criteria: (a) CI users activated for at least six months; (b) complete insertion of the CI electrodes; (c) both genders; (d) aged 18 years or greater; (e) performed the auditory cognitive potential; (f) were aware of the research and signed the informed consent. Exclusion criteria were: (a) patients with orthopedic or neurological restrictions that prevented the performance of the CDP; (b) patients complaining of dizziness or imbalance.

### Casuistic

At the time of the research 111 patients with post lingual deafness were implanted in this service; 60 were <18 years old and were excluded. The remaining patients were contacted by phone and 26 gave positive responses. Eighteen of them agreed to participate in the study. Subjects who declined reported the following reasons: some lived in states with sufficient support for CI recipients; others did not want to participate in this scientific study. Of the 18 individuals who agree to participate, only 14 met the selection criteria and were divided into two groups: (a) Group+ (G+) composed of nine CI users with good hearing performance; (b) Group− (G−) consisted of five CI users with poor hearing performance. All patients were fitted with multi-channel cochlear implants (Nucleus 22, Nucleus 24) activated for at least six months. Complete electrode insertion was controlled by postoperative radiologic studies. For “poor” speech performers, the internal CI unit was tested and retested using the software provided by the manufacturer (Impedance check RA26, Cochlear Corporation – Denver, CO, United States).

For each CI user a control subject was selected, matched for age and education. The control group had 26 subjects, 15 females, with mean age of 44.7 years (range 23–68, SD 14.75). All control subjects had bilateral pure-tone audiometric thresholds of 25 dB HL or better from 500 to 8000 Hz, no hearing or tinnitus complaints, and no history of otological diseases. The exclusion criteria were the same as for the study group.

Good hearing performance was defined as ≥80% speech recognition in open-set sentences. A third group of seven normal-hearing subjects, with normal pure tone audiometry (PTA), speech recognition thresholds (SRT), and speech discrimination scores (SDS), was used as the control group (CG) of the study.

## Computerized dynamic posturography

CDP is an objective and non-invasive test that allows the graphic recording of body position against various stimuli. The CDP is a unique assessment technique used to objectively quantify interactions among vestibular, visual, and somatosensory inputs necessary for balance control, coordination of motor responses, and alignment of the center of gravity (COG). Therefore, CDP analyzes the ability to maintain or regain control of body posture under a variety of sensory conditions. This study used the Smart Equitest System^®^ (Neurocom International Inc. – Clackamas, Oregon, United States). Each of the selected patients performed a dual task test in order to assess short-term memory, which depends directly on proper functioning of the hippocampus. To evaluate the short-term learning ability in the performance of postural recovery strategies, two CDP tests were selected: (1) the sensory organization test (SOT); and (2) the adaptation test (ADT).

The standard SOT protocol consists of three 20-s trials of six different sensory conditions (SOT1 to SOT6) and postural control depends on the interaction between vision, vestibular, and proprioceptive inputs. The subject was instructed to stand as still as possible, with the head erect, eyes looking forward, and hands along the body during specific visual, floor surface, and visual surround manipulations. An equilibrium score based on the magnitude of the COG movements, expressed as percentage of the limits of stability, was calculated for each trial. A score of 100% indicates perfect stability and 0% indicates sway that exceeds the limits of stability or a fall. A composite score (CS), a weighted average of the scores for the six sensory conditions, was calculated and used in the statistical analyses, as well as the results of each individual condition.

The ADT assesses the ability of the automatic motor system to adapt to an unexpected external disturbance. Adaptation is assessed by determining the ability to suppress inappropriate responses to the external disturbance. The test consisted of five trials in which the floor surface was tilted upward (toes up) and five trials in which the floor surface was rotated downward (toes down). Each of the five rotations lasted 400 ms with an amplitude of 8°. The time between the trials was randomized from 3 to 5 s. The ADT score quantifies the magnitude of the anterior-posterior (AP) sway and muscle reactions for body balance control following unexpected support rotations that determine ankle angle changes. The mean scores of toes-up and toes-down sway energy were included in the statistical analyses. Similarly to the SOT, the program provides the results in graphical and numerical scales.

For each patient, both CPD tests (SOT and ADT) were applied as dual task performance on the same day, with a 40–60 min interval between them. Patients were asked to memorize the test conditions and strategies used for body balance control during the first test (FT), in order to smooth and improve the re-test (RT) performance.

### Statistical analyses

Data did not follow a normal distribution, thus non-parametric tests were performed. The Wilcoxon test was used to determine differences between the: (1) individual equilibrium scores across the six sensory conditions (SOT1, SOT2, SOT3, SOT4, SOT5, SOT6); (2) the CS; and (3) the toes up and toes down sway energy scores, between the FT and RT performances measures. The Kruskal–Wallis test and Tukey's multiple comparisons were used to verify the hierarchy of differences between groups. A significance level of 95% (*p* < 0.05) was adopted, according to the standards used in biological studies.

## Results

The average age of the 14 patients was 50.43 ± 14.35 years and of the seven controls 47.57 ± 13.5 years. Gender distribution was similar between all groups. The characteristics of the patients, duration and etiology of the deafness, CI device, and activation time are described in Table 1.

**Table 1:** Clinical and demographic data of study group subjects (*n* = 14).

	G− (*n* = 5)	G+ (*n* = 9)
*Female gender, n (%)*	2 (40%)	5 (56%)
*Median age (min–max)*	57 (34–66)	38 (23–57)
*Duration of hearing loss (years): median (min–max)*	18.8 (1–36)	12.8 (1–43)
*Cochlear implant activation (months): median (min–max)*	20.33 (5–55)	25 (9–37)
*Nucleus 22 processing strategy: speak*	3	2
*Nucleus 24 processing strategy: ACE*	2	7
*Deafness etiology*		
Meningitis	5	0
Trauma	0	1
Otosclerosis	0	2
Ototoxicity	0	1
Chronic otitis media	0	1
Unknown	0	4

G−, cochlear implant users with poor hearing performance; G+, cochlear implant users with good hearing performance; min–max, minimum–maximum; ACE, advanced combination encoder.

Comparing hearing performance in open-set sentences and monosyllabic words among the groups, including free open set and monosyllables discrimination, G+ showed similar results to the CG (*p* = 0.836 and *p* = 0.264, respectively). Both outperformed the G− group (*p* < 0.001 in both cases, Kruskal–Wallis test and Tukey's multiple comparisons). The results show that the hearing performance and word discrimination of G+ after CI surgery were similar to CG subjects (Table 2).

**Table 2:** Speech recognition in open-set sentences and monosyllabic vocal discrimination of study and control group subjects.

Groups		G+	G−	CG	Kruskal–Wallis test (*p*)	Tukey's multiple comparisons (*p*)
Open-set sentences	Median (min–max)	100 (80–100)	0 (0–20)	100 (100–100)	0.004^a^	G+ = CG > G− (<0.001)
Monosylabic vocal discrimination	Median (min–max)	84 (58–100)	0 (0–88)	100 (96–100)	0.001^a^	G+ = CG > G− (<0.001)
*n*		9	5	7		

G+, cochlear implant users with good hearing performance; G−, cochlear implant users with poor hearing performance; CG, control group; min–max, minimum–maximum.

a Statistical significance level.

In the first CDP test, both G+ and G− had similar performance in all six sensorial conditions, but underperformed CG, whose results were significantly better in SOT3, SOT4, SOT5 and SOT6 and CS (Table 3). Comparing the dual task performance on CDP and the weighted average between all tested conditions, the G+ group showed a significantly better performance at RT in SOT4, SOT5, SOT6, and CS, which was not observed for G− and CG. In the CDP retest, G+ outperformed G− in SOT5, SOT6, and CS, suggesting more effective learning in G+ as compared to the other two groups (Table 4).

**Table 3:** FT results according to groups.

Groups		G+	G−	CG	Kruskal–Wallis test (*p*)	Tukey's multiple comparisons (*p*)
SOT1 FT	Median (min–max)	94.3 (91.3–95.7)	93 (92–97.7)	96 (92.3–97)	0.172	G+ = G− = CG
SOT2 FT	Median (min–max)	92.7 (86.7–94.3)	90 (88–96)	94.7 (90–97.7)	0.088	G+ = G− = CG
SOT3 FT	Median (min–max)	91 (79.7–95.3)	90 (82.7–91.3)	94 (93.3–97)	0.018^a^	G+ = G− < CG (0.036^b^)
SOT4 FT	Median (min–max)	68.7 (55.3–83)	81.7 (29–85.7)	87.7 (83.7–93.3)	0.003^a^	G+ = G− < CG (0.039^b^)
SOT5 FT	Median (min–max)	47.7 (0–61.3)	0 (0–25.7)	68.7 (59.3–76)	0.001^a^	G+ = G− < CG (<0.001^b^)
SOT6 FT	Median (min–max)	4.3 (0–66.7)	0 (0–7.3)	66 (20–82.3)	0.003^a^	G+ = G− < CG (0.001^b^)
CES FT	Median (min–max)	58 (42–77)	51 (37–58)	81 (69–88)	0.002^a^	G+ = G− < CG (0.001^b^)
*n*		9	5	7		

G+, cochlear implant users with good hearing performance; G−, cochlear implant users with poor hearing performance; CG, control group; min–max, minimum–maximum, CDP, computerized dynamic posturography; SOT, sensory organization test; CES, composite score; FT, first test.

a Statistical significance level.

b CG showed significant higher scores as compared to G+ and G− in SOT3, SOT4, SOT5, SOT6, and CES conditions.

**Table 4:** RT results according to groups.

Groups		G+	G−	CG	Kruskal–Wallis test (*p*)	Tukey's multiple comparisons (*p*)
SOT1 RT	Median (min–max)	94 (90.3–95.3)	94.7 (90.7–95)	95.7 (94.7–97.7)	0.005^a^	G+ = G− < CG (0.012^b^)
SOT2 RT	Median (min–max)	90.3 (85–94.3)	89.3 (83–92)	94 (90.7–97.7)	0.012^a^	G− < CG (0.010^b^)
SOT3 RT	Median (min–max)	89.3 (76.3–95.7)	90 (80–95)	93.3 (92–97.7)	0.076	G+ = G− = CG
SOT4 RT	Median (min–max)	82 (64.3–88.3)	85.7 (40–90)	88.3 (85.3–95.3)	0.023^a^	G− < CG (0.050^b^)
SOT5 RT	Median (min–max)	64.3 (0–78.3)	0 (0–29.3)	74.7 (59.3–80.3)	0.007^a^	G− < G+ < CG (0.001^b^)
SOT6 RT	Median (min–max)	27.3 (0–70.7)	0 (0–0)	77.3 (38–81.7)	0.001^a^	G− < G+ < CG (<0.001^b^)
CES RT	Median (min–max)	66 (48–82)	52 (39–58)	83 (72–89)	0.001^a^	G− < G+ < CG (<0.001^b^)

G+, cochlear implant users with good hearing performance; G−, cochlear implant users with poor hearing performance; CG, control group; min–max, minimum–maximum; CDP, computerized dynamic posturography; SOT, sensory organization test; CES, composite score; RT, re-test.

a Statistical significance level.

b CG showed significant higher scores than G− in SOT2 and SOT4. CG outperformed G+ and G− in SOT5, SOT6, and CES (CG > G+ > G−).

In the ADT dual task performance, when comparing toes up and toes down sway energy means scores, no statistical difference between the groups could be found (*p* = 0.616 and 0.563, at both tests conditions, Kruskal–Wallis test and Tukey's multiple comparisons).

In the P3 test, this study observed significantly longer P3 latencies among G− as compared to G+ and CG for the 2000/1000 and 1000/1500 Hz test conditions (*p* = 0.007 and *p* < 0.001 for the former; *p* = 0.001 and *p* < 0.001 for the latter test), but no difference between G+ and CG (*p* = 0.177 and *p* *=* 0.601) (Kruskal–Wallis test and Tukey's multiple comparisons), as shown in Table 5.

**Table 5:** P3 latencies in the 2000/1000 Hz and 1000/1500 Hz test conditions according the study group.

		G+ (*n* = 9)	G− (*n* = 5)	CG (*n* = 7)	Kruskal–Wallis test (*p*)	Tukey's multiple comparisons (*p*)
P3 latency 2000/1000 Hz	Median (min–max)	351 (327–450)	423 (399–492)	342 (261–351)	0.006^a^	G− > G+ = CG (<0.001^a^)
P3 latency 1000/1500 Hz	Median (min–max)	345 (330–474)	457.5 (450–501)	351 (308–390)	0.023^a^	G− > G+ = CG (<0.001^a^)

G+, cochlear implant users with good hearing performance; G−, cochlear implant users with poor hearing performance; CG, control group; min–max, minimum–maximum.

a Statistical significance level.

## Discussion

The CDP is an objective, non-invasive test. Dual task performance was chosen, with a brief interval between them to assess short-term learning and memory ability as part of postural recovery strategies. The choice of the interval between each performance allowed a rest period of about 40–60 min. Thus, the patient could recover from muscular effort during FT to ensure that fatigue would not interfere during RT, so the RT result would reflect only the short-term memory ability.^10^ The CS improvement during RT is expected for those subjects who have normal short-term learning ability and proper hippocampus function.

The small sample size results from the restrictive selection criteria: only patients with full CI electrode insertion were included, in order to eliminate surgical bias. Age and CI activation time were used as group pairing variables, because it is known that both could change SOT performance and P3 latencies.^11^, ^12^

The better RT performance of G+ in SOT4, SOT5, SOT6, and CS suggests that CI subjects are able to “learn” the presented sensory conditions in the FT and use this learning and memory ability during RT. This ability comes from an accurate CNS sensory input integration, responsible for motor strategies to assure body balance control.^10^ Thus, these subjects actually have an accurate short-term memory and learning ability. The difficulty of the G− patients could be attributed to functional impairment of structures involved with speech discrimination, stimulus recognition, and learning skills, abilities required to generate appropriate motor responses for postural correction as well as stabilization and balance control. Considering that CG showed a greater FT response, a significant improvement in RT was not expected, which is in agreement with the results (Table 4).

Hearing provides acoustic information about the environment, enabling us to notice and avoid environmental hazards that may lead to a fall.^6^ Studies testing the associations between hearing acuity and postural balance are scarce and show conflicting results. Occupational health studies have shown a correlation between noise exposure and impaired postural balance.^13^, ^14^ Studying older female twins, Viljanen et al.^6^ showed that poor hearing acuity increased the risk of falls when compared to sisters with good hearing acuity, which could be partially explained by their poorer postural control. The present study observed longer P3 latencies in CI users with poor hearing performance, as compared to CI recipients with good hearing performance and controls. These findings indicate that hearing impairment may directly or indirectly interfere in cognitive tasks evaluated by the P3 test. The same group G− showed a worse CDP performance compared to the other groups. Postural stability during static and dynamic tasks could be described as the ability to modulate postural response magnitude, thus generating an accurate motor response. It should be remembered that postural control comes from and relies on the interaction between somatosensory, vestibular, and visual systems, and that postural imbalance increases with increasing number of underlying affected systems. Although the auditory system is not usually cited and included in central processing integration of peripheral inputs involved in postural control, these findings suggest a direct or indirect influence of hearing, demonstrated by the worse SOT results of G− with poor auditory performance.

Toes-up or toes-down tilts of a platform on which a subject stands induce early responses of the leg muscles stretched and late responses in the antagonist muscles. The support surface is slowly rotated about 8 degrees while the body is stationary. The rotational movement of the platform triggers an automatic postural response as defense against the postural disturbance. According to Nardone et al.,^15^ the origin of the late responses in the antagonist muscle comes from the overall postural imbalance and the postural set. Thus, it does not correlate with or depend on cognition. When analyzing ADT dual task performance results, it was not possible to find a statistical difference comparing FT and RT among the groups. Since ADT is used to investigate the automatic postural control, memory and cognition are not required for this task. Therefore, diverging results between groups were not expected as were during SOT conditions.^15^

Long-latency components of event-related potentials (like the P3) correlate with the ability of subjects to detect and process unexpected, novel, or task-relevant stimuli. Task-relevant late positive components can be recorded in the neocortex and hippocampus while performing an auditory discrimination task.^16^ The cortical potential elicited in the context of auditory target detection tasks includes the N1, P2, and P3 components. The concordance between magnetoencephalographic (MEG) and brain electric source analysis (BESA) source localization supports the notion of generators in temporal lobes for the N1/P2 complex and generators in temporal and hippocampal areas for the P3 component.^17^ Thus, as literature reports, CNS structures functionally involved with explicit memory give rise to auditory, visual, and somatosensory central integration, and can be localized in the hippocampus and temporal lobe.^16^, ^17^ In fact, Viljanen et al.^6^ have already reported hearing loss as a predictor of falls and poorer postural balance control. In their study,^6^ pure tone thresholds were reported without speech reception thresholds. Therefore it is possible that the higher risk of falls in this sample may not be related to hearing loss itself, but rather to a lack of sensory input or cognitive impairment.

This study had some limitations. First, from the initial sample of 111 eligible patients, only 14 were included in the study group. As mentioned, this is the result of the rather strict selection criteria (*n* = 60 were under age 18), and the fact that 25 subjects could not be contacted by phone and that some lived too far away to enter the study. Only five patients had poor hearing performance, reflecting the selection criteria for CI surgery for post-lingual deaf patients at this center and indicating that almost all CI surgeries with complete electrode insertion were successful to establish useful hearing and speech discrimination. Due to the strict inclusion criteria a small number of subjects in each group were expected, reducing statistical power, so more studies including patients from multiple CI centers should be conducted to overcome this limitation.

It is not possible to affirm that the findings are exclusively related to auditory and/or vestibular neural pathway impairment, because all G− patients were deaf due to meningitis. Besides causing deafness, meningitis can also affect several other functional and anatomical CNS areas.^2^, ^18^, ^19^ As the goal was to evaluate postural recovery in CI users, G+ and G− groups had to show distinct auditory performance, so etiology of hearing loss was not equally distributed among both groups. Neither can it be claimed that the results arise from learning and memory network involvement. Thus, further studies are necessary to clarify these questions.

According to the results, postural recovery appears to be related to learning and memory, as well as to speech and auditory performance. It is also possible that a compromised network interferes in both auditory performance and learning strategies necessary for body balance control. If proven to be effective, the relationship between auditory and vestibular pathways opens a new research field, and strengthens the role of cognitive auditory potentials and speech discrimination tests as predictors of postural performance and vestibular rehabilitation.

## Conclusions

Comparing the dual task performance in CDP and the weighted average between all tested conditions, cochlear implant users with good hearing performance showed better re-test results in SOT4, SOT5, SOT6, and CS, and also had a higher index of postural recovery, as compared to CI users with poor hearing performance. Cochlear implant users without good hearing performance had significantly lower levels of short-term learning ability than the other two groups in SOT5, SOT6, and CS.

## Funding

Funded by Conselho Nacional de Desenvolvimento Científico e Tecnológico – CNPQ.

## Conflicts of interest

The authors declare no conflicts of interest.
